# A Multi-Collaborative Ambient Assisted Living Service Description Tool

**DOI:** 10.3390/s140609776

**Published:** 2014-06-03

**Authors:** Jorge L. Falcó, Esteban Vaquerizo, José Ignacio Artigas

**Affiliations:** Grupo de Investigación Tecnodiscap-welltech, I3A, Universidad de Zaragoza, María de Luna 1, Zaragoza 50018, Spain; E-Mails: evaquerizo@unizar.es (E.V.); jiartigas@unizar.es (J.I.A.)

**Keywords:** Ambient Assisted Living (AAL), multidisciplinary, assistive technology, collaboration, sustainability, services provision

## Abstract

Collaboration among different stakeholders is a key factor in the design of Ambient Assisted Living (AAL) environments and services. Throughout several AAL projects we have found repeated difficulties in this collaboration and have learned lessons by the experience of solving real situations. This paper highlights identified critical items for collaboration among technicians, users, company and institutional stakeholders and proposes as a communication tool for a project steering committee a service description tool which includes information from the different fields in comprehensible format for the others. It was first generated in the MonAMI project to promote understanding among different workgroups, proven useful there, and further tested later in some other smaller AAL projects. The concept of scalable service description has proven useful for understanding of different disciplines and for participatory decision making throughout the projects to adapt to singularities and partial successes or faults of each action. This paper introduces such tool, relates with existing methodologies in cooperation in AAL and describes it with a example to offer to AAL community. Further work on this tool will significantly improve results in user-centered design of sustainable services in AAL.

## 1. Introduction

This paper describes a multidisciplinary collaborative tool for service description in Ambient Assisted Living (AAL). Zaragoza Ambient Assisted Services Description Tool (ZAAS-DT) is the result of direct experience in research projects. Its concrete origin comes from December 2008 when the MonAMi [1] project had a strict requirement from European Commission evaluation to make a description of project services in a way that involved stakeholders groups inside the project could share information and understanding. Coordination of national demonstration centers developed quickly a description tool that was a deliverable (D10.2) and proved useful and successful within the project context. D10.2 was written in response to a request at the MonAMi second annual review for an integrated view of service description, a clear mapping onto technology modules to be used and design of services that could prove the utility of a technological core platform inside MonAMi consortium. Later it has been used to overcome several difficulties of collaboration and common decision taking among different actor profiles, mainly technology and user-centered researchers and to a minor extension institutions and companies interested in bringing the product to the market.

This tool (not a software program, basically a methodology or information structure and way to display it) has evolved during its years of use from 2008 to 2014, and also through the interaction of students of three different master degrees: Social Gerontology (University of Zaragoza), once a year, whose main profile are caregivers, nurses and medical doctors; Master in Electronic Engineering, (University of Zaragoza), once a year, whose main profile are engineers (industrial and telecommunication); Master in Personal Autonomy (University of the Basque Country), once every two years, with heterogeneous profiles: psychologists, engineers, nurses, elderly care-givers, *etc*.

Former works started to identify this need. Ambient Intelligence (AmI) vision described by Weiser [2] started AmI paradigms. From the field of technological solutions for people with disabilities there have been identified the need for integration of heterogeneous technologies in a seamless way and easy to use by a non-technological user [3]. In recent years AAL activity has faced the need to coordinate the knowledge and methodologies of different disciplines, for it is a field in which technology comes specially closer to human being everyday life and way of living, implying understanding of preferences, needs, applications, and moreover needing new services to become real in a business, economical and institutional complex world where non-written rules are largely influencing the success and sustainability of any service. It is also a very down-to-earth field, in which necessity becomes the leading parameter of developments very much linked to human global paradigm.

Service description in AAL has proven to be a point of issue when several disciplines must participate in designing, developing, deploying and/or evaluating AAL services, especially when taking decisions that directly affect the interface and functionality with the user, and sustainability in an institutional and economic background.

This work summarizes a structured solution built up during several research projects in AAL involving different disciplines, highlighting key issues to be taken into account and supporting their relevance with direct experience in development projects. We have identified four main disciplines collaborating for the success of a supporting system for dependency and elderly: (1) technological; (2) social and user's experts; (3) market and company experts; (4) institutional representatives, for political instances proved to be key actors in the deployment of real and sustainable solutions in different countries during the MonAMi project.

Other projects which experience is condensed in this work are Ashored (1992), SCALP (1994), and some recent as AmiVital (2006), Siamyd (2011) [4], and direct collaborations with user organizations in counseled flats and with health institutions in particular flats. Experience has been gathered from all of them, and the recent ones have already been working internally with this tool, proven useful and easy-to-use. Some identified trouble spots are:

*Vocabulary*: the word “service” for a technological professional often means a piece of code that applications use for their own performance, as in a printing service in a computer or communications services able to send alerts through telephone or internet. For a social worker, “service” is a unit of support ready to be used or given to a person, in this case in a frailty situation. This has consequences in the expected maturity of the service as such, a technological minded-people may think a service is ready for transference when other applications can use it and for a social minded person a service is not ready until it can be delivered to the objective person of the action.
*Stating service requirements*: We have found technological and user or social minded people find it difficult to adapt the way of expressing requirements. As an illustrating example, for technological people it would be correct to indicate “a luminous spot of about such dimensions” or “large enough to be clearly appreciated by an elder”, while for a non-technological people this is the same as “a small light bulb”. In this case, division among functional and technological specifications has given good results, although some training is needed in both sides. It also has proven useful to add accompanying information about the supporting reason for each requirement, date of such information and the source, so it can be reviewed if technological implementation may offer alternatives. Cross-related tables with the requirements and their solutions have proven a significant advance in common understanding and collaborating tool.
*Keeping user requirements in the design loops and cases of use*: Many works take advantage of cases of use as a particular way to express user requirements. Normally these are filled in by users-social oriented people, and digested by technological people, and we have found a lack of feedback at this point, when some design decisions need to be made due to budget, technological availability of solutions of others. We have found necessary to close the circle to show user oriented workers the consequences of each modification, being accompanied by budget consequences or restrictions. In this way, both groups of workers can understand the consequences of each technical or budgetary decision, and participate with knowledge in them.
*Enhancing understanding of components of a service and their implications, i.e.*, for taking steering decisions.
*Maturity and unexpected results*, as implications of delays or drop-offs: in some research projects timing is not working at the end of the day as planned in the very beginning, and timetable exerts dependency pressure on the different tasks to be performed. To evaluate user experience deployment a minimum maturity of the development is required. When problems appear in a development, non-technological people find it difficult to understand which consequences this may have, and which functionalities may be compromised. So at the time of adapting the project to its reality, this information understanding is critical so each specific work-group find grounds and understands others positions to get to some decisions by consensus or at least with equilibrium by all parts, which in our experience means to decide which changes to do in functionalities, number of people to participate in evaluation, duration of evaluation, overall cost… or which services are to be promoted or dropped-off.
*Information flows*: Cases of use normally have associated information flows, that come in a sequence. For technological minded professionals it is normally easy to foresee which information is going to flow at which stages. This is not so with non-technological people, so a clear and graphical description of the system moduli and how information is flowing among them and users has proved efficient. This also capacitates non-technological professionals to participate in design decisions with direct knowledge of the system, so decisions are taken with better information background.
*Relevance of each module in global performance*: Relation among technological challenge, expected final cost, need of supporting infrastructure and impact of service performance on the user experience is critical when deciding which functionalities should be accomplished by project services and which technological implementation is made to support them. In several projects a pool of different possibilities is generated, and then selection is needed. Equilibrium among each component is critical, being an important factor the final user experience. Information has to be made permeable to each deciding discipline, to understand the implications on neighboring disciplines and arrive to a consensus. For that, service description is the common pool where all relevant features are to be included and made understandable for each different discipline participant.
*Service contextual information as critical for sustainability*: From a technological, company and institutional point of view it is necessary to know which services and technological infrastructure must be deployed. Sometimes this is a matter of getting the appropriate permissions, sometimes it is necessary to add such technological infrastructure and it must be maintained later one. From a user point of view, there are other services that may enter in conflict (several alert and tele-alarm services) or may collaborate for the benefit of the user (surveillance service infrastructure already existing as tele-alarm service centers, relatives or neighbors support, services of delivering prepared foods that may simultaneously perform other tasks, *etc.*). Moreover, local needs have been found to be dependent on geographical and cultural differences, for each society is used to supporting people in different ways. Macroscopic studies give an overall vision that lacks information of local singularities, so contextual information relevant for sustainability is necessary to adapt sustainability to the different regions of interest.

## 2. Related Works

We have found no such service description tool in the bibliography, although there are some partial approaches that describe some moduli of the perspective offered in this work. Many of them center the focus on the technology-user interaction, searching for user involvement in the design and evaluation processes and retrieving a good set of user requirements. This is a very important issue and the one that is probably more frequently addressed. Our approach allows integration of any of those methodologies for user interaction and validation, centering the goal in a wider information structure to share understanding of service in AAL not only between designers and user interaction, but also with market oriented professionals, society associations and institutions. Innovation respect to those communications is basically the identification of the needed information bits not only between users and technologists, but also with institutions, society and commercial or marketing sector. From that first step and based on experience, this work builds up the methodology as a scheme for service description in a way that we have found understandable by different professional sectors.

In recent years there have been many AAL projects and works that have faced the need to coordinate the knowledge and methodologies of different disciplines. Several examples show this is partially covered in literature, both in AAL and in health services, mainly promoted by the interoperable architectures currently developed and tested, and especially in the areas of rising user requirements in the design process.

Several AAL works are reported to attend user needs and contextual information. Most, though, do it without a reference methodology to raise and keep specifications on track and centered basically in technological solutions. Approximation about contextual information is basically technological, to try and have different pieces of technology understanding and empowering contextual information.

In [5] the authors first describe the technological architecture, being afterwards explained how user needs are met, not showing any specific modulation of technology based on user interest or taking into account other disciplines mentioned in this paper. Another example is [6], in which Unified Modeling Language (UML, a modeling and specification language used in software engineering) is used developing an interesting concept of famiWare, which gives a good description of contextual information and handling. They center on technological challenges showing user needs as something already decided or put into value. This paper also shows how the use of UML is very adequate to this type of approaches, for they remain the focus in the technological structures and their functionalities.

A similar work is [7], which clearly states the functional goals to be met in the authors' focus, giving good reasoning of relevance of such topics and making them the starting point of their development. No methodology or process is shown or mentioned to integrate ideas and perspectives from different disciplines, nor to keep them aware through the project, which probably was done anyhow, may be in a non-structured way, for this work describes evaluation by several non-technological disciplines. Scenario methodology is implicitly shown and structured evaluation with users reported. Description of interaction among several disciplines involved is not shown, further than the evaluation process in which users are involved. Similarly, in [8] user needs are taken into account focusing them on the technological aspects and solutions, no methodology is shown in user needs nor evaluation.

Several other works include some referenced methodology for user requirements. In [9] work in the health field with case-study methodology is described, summarizing in two case studies all their goals (long term monitoring and smart hospital). Modular schemes describing service components are similar to the ones developed in our integrated service description tool, adding component interactions that give an approximation of information flows, although it seems oriented to technological people, not so much understandable for non-technological profiles.

Based on user needs and with the aim of improving alert handling, [10] does have a representation of functionality as an algorithm description, and contextual information is also displayed. It does have two case studies with which requirements are validated and their argumentation explanation is shown and applied. Degree of integration of activities is very good, but still does not offer some kind of overall tool as we have found necessary.

Many of the literature works referencing some methodology come from software formalization, specifically Service Oriented Architectures (SOA). In [11] the authors provide a complete view of the experiences gained in the MPOWER (IST 034707—Middleware Platform for eMPOWERing cognitive disabled and elderly) project with respect to using model-driven development (MDD) techniques for Service Oriented Architecture (SOA) system development in the AAL domain. Method used is to investigate and record the user needs, define a set of reusable software services based on these needs, and then implement pilot systems using these services. Further, a model-driven tool-chain covering key development phases was developed to support software developers through this process. Evaluations were conducted both on the technical items (methodology and tools), and on the end user experience from using the pilot systems at trial sites. It shows collaboration has taken place. It also introduces HL7, a methodology for a very clinically oriented service description used in health records [12]. This methodology does provide a use case diagram basically with components; it also provides some UML description of functionality in a algorithm way, thus being the most complete of the bibliography findings. Still, it is technologically oriented, and very complete as far as medical history and health record for classifying profiles of users, which in AAL would have an equivalent in the history of a particular user, but not so much in the service description aspect. It focuses a lot on describing the patient profile and the progress of the service itself.

Also from the field of software engineering, there is related work done on non-functional parameters. A good example is a review of the validation of methodology for non-functional features—response time, availability, cost, reliability, throughput, successful execution rate, reputation and accuracy—found in [13]. This work also shows that the primary studies do not present alignment with any standard model, apart from one which is aligned with ISO/IEC 9126 Standard Quality Model [14]. Despite this lack of alignment, most of the attributes that have been identified in the selected primary studies can be mapped to the same characteristics that are specified in this standard.

In [15] the authors show a requirements engineering method, thus covering the part of arising requirements. This very interesting work focuses on features that are non-functional themselves, specifically background features of ubiquitous systems. As a potent software design tool, it gives complete meta-models which show the interdependency for the programmer, in a UML style, designed for technological developers. In a similar fashion [16] shows aspects associated to the generation of code from requirements, it also includes validation procedures, and provides links among components directly oriented to technological people. It is based in many tables and UML-like diagrams with the corresponding interrelations. These works lack provision of understandable information of insight of service components and interrelation for plain users. Again, similarly [17] offers a methodology for supervising non-functional features of AAL services, a verification approach based on timed traces semantics and a methodology based on UML-RT models (MEDISTAM-RT) to check the fulfillment of non- functional requirements, such as timeliness and safety (deadlock freeness).

Related with the item of taking steering decisions, also in the field of software developers and including non-functional parameters, [18] gives metrics-like procedures for service selection as far as Quality of Service and other parameters such as cost or will to pay, that are needed for service selection, supposed that a pool of such services is available.

A very interesting review in this topic, centered on the Service-Oriented Architecture (SOA) paradigm, is the work of Alves *et al.* [19] which reviews the use of methodologies and concludes that evidence for adoption of the methods is not mature, given the primary focus on model examples. The proposed approaches still have serious limitations in terms of rigor, credibility, and validity of their findings. Additionally, most approaches still lack tool support addressing the heterogeneity and mostly textual nature of requirement formats. Further empirical studies should be performed with sufficient rigor to enhance the body of evidence. In order to address the scalability and popularization of the approaches, future research should be invested in tool support and in addressing combined software product line engineering (SPLE) adoption strategies.

A singular study to be highlighted in this regard is [20], which proposes the use of 2001 International Classification of Functioning, Disability and Health (ICF) taxonomy from World Health Organization [21], concluding that AAL is too technology-based, even when stating that works are user-centered, which is one of the identified origins of the need of further collaboration tools. This paper proposes an ICF-based taxonomy to evaluate contextual factors influencing efficiency and acceptance of AAL services.

Still unsolved, methodologies that use a common description tool for all stakeholders implied, summarizing technological, functional and non-functional information in an way accessible for non-technological professionals to promote the involvement of all stakeholders in the decisions and design and validation processes are critical to redirect technological solutions to fit in the new AAL paradigm in which sustainable and efficient services can be provided to support independent and healthier lifestyles for people in frailty situations. It is clear that “these technologies have the potential to affect profoundly, both positive and negatively participants meaning and experience of the home environment” [22].

There are many referenced methods to address both user characteristics description and classification and validation of accessibility and good user interaction. This work is not centered on those methodologies, there are many and some are proven efficient. Moreover, our work can take advantage of them. In our case we have used mainly user-fit [23]. There are others such as persona [24–26] which could be integrated in the section of user-needs and interaction of our methodology or exchange information structure. A similar project to MonAMi is UNIVERSAAL [27], which promotes an open platform for AAL service delivery and discusses users' involvement. Accessibility and Usability Validation Framework for AAL Interaction Design Process (VAALID) [28–30] focuses in developing an integrated development environment, VAALID Integrated Environment (VAALID IDE), for computer-aided design and validation of User-Interaction subsystems that improve and optimize the accessibility features of Ambient Assisted Living services, for the social inclusion and independent living of senior citizens. It is done with a 3D-immersive simulation platform so it facilitates the user-interaction system design. We can also take advantage of other works whose aim is to validate a good accessibility or user interaction, like the VERITAS project [31].

## 3. Experimental Section

### 3.1. Basis of the Service Description Tool

The service description tool has to include four main groups of features, related with the four relevant types of stakeholders:

‐ Functional features linked to user requirements, to allow for revision when changes are needed. This also includes user profiles, stakeholders and secondary users, implied scenarios and activity analysis. User-fit, Persona and cases of use are common structured methodologies used in this regard. We refer to these items as user-field description.
‐ Service description as far as moduli, components, information flows with special emphasis in user information. Also the interrelation among components, and which one is related or dependent on which other. It may also include user-interfaces description, and form-factor of devices to be used. We will refer to this group as technological-field features.
‐ Maturity and cost of technology in a market-way reference. This also includes potential developers, financing schemes and value chain. We refer to this group as market-field features.
‐ Institutional and wide context description or ecology of the service. Technological infrastructure needed, human or other services infrastructure in which find support, conflicting services in the community, legal and ethical issues as understood locally and globally, institutional interest and public economy implications. We will refer this as ecology-contextual features, including social, institutional and service supporting structures.

This information has been gathered in the mentioned research projects and used as a common tool, progressively integrating demanded information as scope of the overall efficiency and sustainability of services was considered in real demonstrators: a shelter home with 15 installed rooms, three private flats with connection to relatives and health center, an independent living training flat of ATADES (Spanish Users Association for Cognitive Disability), and some small demonstrators in special education schools located in Zaragoza (Spain).

Integrating needs, pieces of information and ways to display it to have it comprehensible has been performed by authors working with different teams. A critical aspect of this tool is that it has to be efficient as a repository of information, where each interest group may integrate information considered relevant and has to be sure it is described in a way that all the other interest groups understand it.

### 3.2. Relation and Special Interest in Items in Zaragoza Ambient Assisted Services Description Tool (ZAAS-DT)

**Figure 1.**
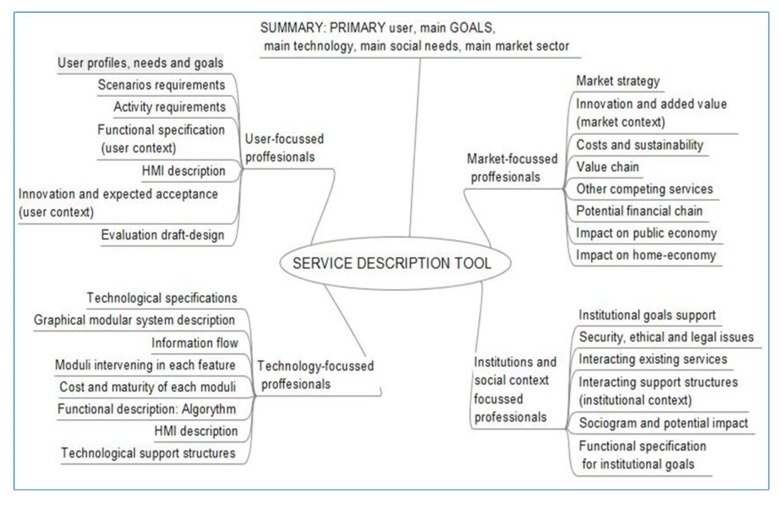
Conceptual map of service description with items or info-bits generated by each professional group.

On the upper part of Figure 1 there is a summary with goals, main users and main system description. Goals to design and define evaluation will also be extracted. This is a large piece of work that doesn't fit in this paper, so we leave it for future communications. It can also be considered as part of our tool.

There are several information items that have been found of special relevance in our experience. They constitute the index of the tool, as shown in Figure 1. Now we go on to describe which of those items have been found of specific interest for other collaborating profiles in our experience. The active role played in our research projects has been traditionally largely played by user-centered groups and technology-oriented groups, so we understand that relation among them is shown in deeper scope. This Service Description Tool should be completed by others who can integrate their experiences more profoundly from the market and social and institutional perspectives.

As all information bits have some interest for all professional groups, we have marked three categories of intensity in this interest: First is the arrow highlighted with wider line, which are commented in the tables and text below. Then is the thinner line, which shows an important interest, although not as critical as the former for the success of the project, in our past experience. Then no line doesn't mean no interest, instead it means the interest has a lower grade than the two former categories, again according to the experience of the projects mentioned.

#### 3.2.1. Information Generated by User-Oriented Professionals

**Figure 2.**
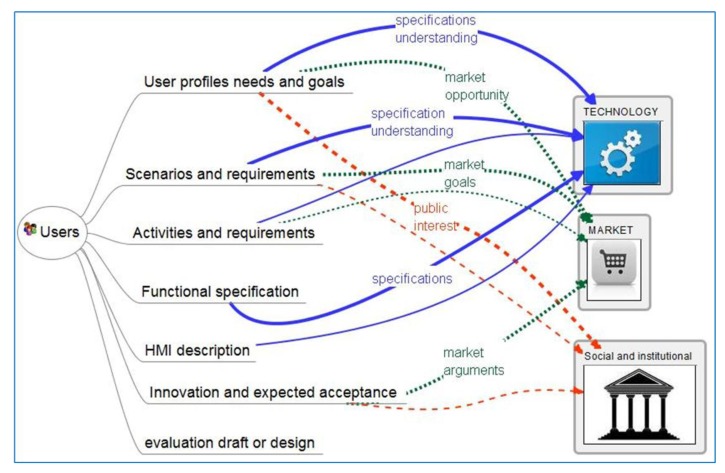
Cross relation of information-bits generated by user-oriented professionals among different professional groups.

User-centered professionals generate basically user needs, preferences and acceptance inside user context, which includes user capacities according to the different profiles, scenarios and activities. This is shown in Figure 2 and detailed in Table 1.

Information is summarized in functional specifications, in which detected conflicts are already sorted out applying preferences and priorities, for different users may have different and conflicting needs (*i.e.*, in a family an elder may need very simplified user interfaces while youngsters may require complete functionality and complexity is not a problem for them). Together with goals, they set the basis for evaluation drafts. Another bit of information found of special interest is user expected acceptance and willingness to have specific features (*i.e.*, willingness to buy).

#### 3.2.2. Information Generated by Technology-Oriented Professionals

**Figure 3.**
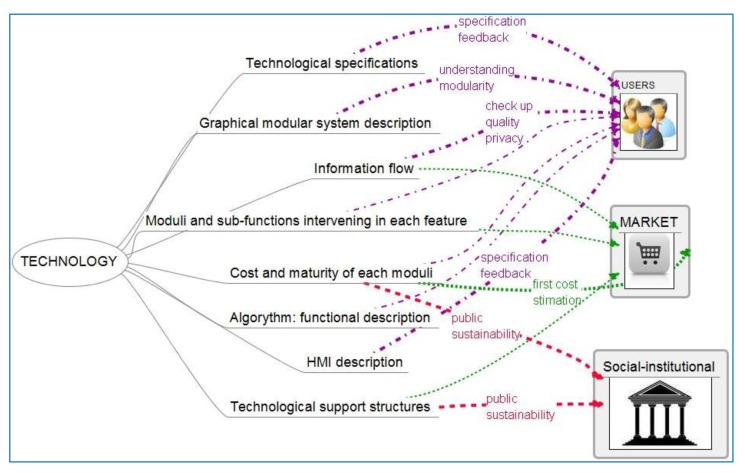
Cross relation of information-bits generated by technology-oriented professionals among different professional groups.

Information generated by technology-oriented professionals is shown in Figure 3. There is a translation of functional needs into technological specifications. Technology-oriented professionals will interact with user-centered ones asking for deeper information about context and needs, and offering different technological solutions with different performance and cost implications. Technology-oriented people ask users to specify functionalities apart from technological examples.

Frequently they have to translate their specifications and should verify that information is not altered by their translations: this is critical in this design phase. As an example, in a moment in our laboratory, user specifications came in the way of “a light bulb of approximately 3 cm, bright and with selectable color”, and technicians have translated “light bulb” into “a luminous element” for it could be a Light Emitting Diode (LED) of any other luminous-generating solution.

Highlighted relations are explained in Table 2. An important finding in sharing this process of initial specifications is to have as soon as possible a dummy prototype or element that gives enough information about how the technical solution will look like and perform. As an example, in a special education classroom we went with different LEDs to check that functional specifications were understood, and found that luminance had to adapt to daylight. A new functional specification was generated as “luminance has to adapt to environmental light”, which translates into technical specifications as the inclusion of an ambient light sensor and generation of some amount of luminance beyond the ambient light.

Moduli understanding is critical for decision taking and understanding where critical information paths have to be addressed, both for quality of service and for privacy issues. From technology previsions cost related to products can also be calculated, which is of critical interest for users, and especially for market and institutional and social context.

Technological support structures include telecommunications, such as WiFi and 3G which are not equally reliable and often rely on external services with which the project administration has to reach agreements and/or modify conditions.

#### 3.2.3. Information Generated by Market-Oriented Professionals

**Figure 4.**
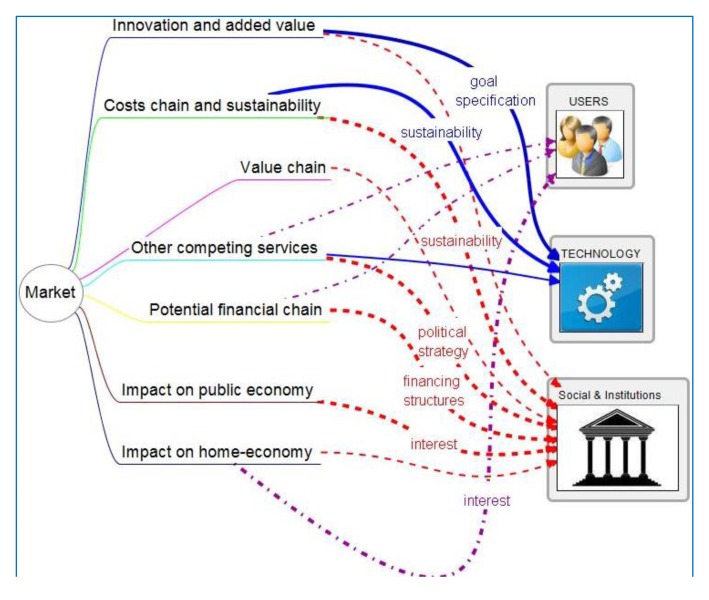
Cross relation of information-bits generated by market-oriented professionals among different professional groups.

Market professionals design market strategy and find strong and weak points in a potentially competing sector. They also study innovation features and acceptable costs, which are fed into specifications and will reorient technology designs. Specific information items to share are shown in Figure 4. Highlighted relations are detailed in Table 3. Analysis of value includes costs, value chain of service provision and study of other existing services that may compete or collaborate. This process has found solutions that made sustainable a service in cases where it appeared difficult or non-sustainable, by identifying synergies with other services, infrastructures and resources. Impact on public economy is critical. This framework has been set by politicians need to choose among alternatives where cost is a must.

Domestic economy impact is also critical for service sustainability, for users may have different alternatives and economy and quality of life are parameters carefully considered when taking assistive decisions. Regarding market distribution and value chain market professional's advice about alliances or usage of support structures that make easier the introduction and sustainability [32].

#### 3.2.4. Information Generated by Social and Institutional Context-Oriented Professionals

**Figure 5.**
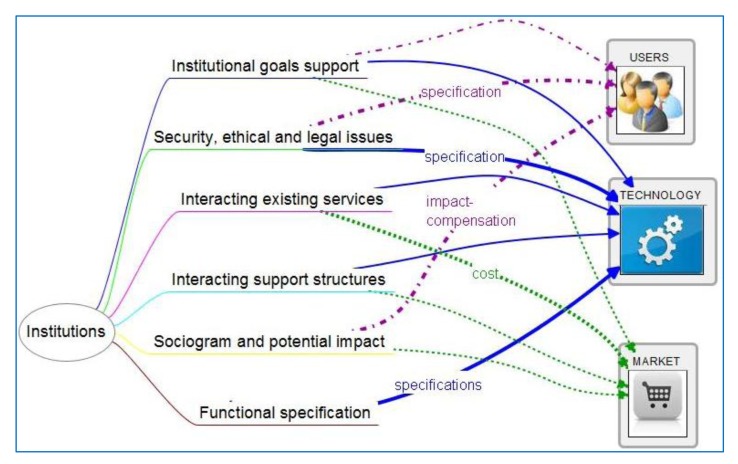
Cross relation of information-bits generated by institutions and social context professionals among different professional groups.

Information generated by the social community is very extensive and diverse. It is, or should be, supported and guaranteed by social institutions. In this term we include user associations and governmental departments and municipalities. Information generated by them is shown in Figure 5 and detailed in Table 4.

It is the ground where real needs take priority or not, as felt by communities and organized by them, so it becomes the real ecology of services, where they not only have to survive, but they need to prove useful for the structures, effort and money invested. They also have to prove ethical and be in line with population felt needs. In the users dimension we consider the small community directly affected by the service, frequent beneficiaries, family, care-givers. By community we mean the collective that forms a wider society cell, neighborhoods, towns, *etc.*, in which people have a common feeling of what issues should be taken care of in particular and which others should be communal.

A major concern about technology found through the projects is data protection. There are many codes of good practices to guarantee ethics, security and data protection, so communities have made rules that especially designers have to take care of. Evaluation design also has be aware of them, to be careful and respect data protection, ethical treaties and avoid historic uses of users as experimental units.

Another critical issue found is the real ecology of the services. The community has services for leisure, business, and domestic activities like buying, cleaning and care taking of communal goods, food delivery and special care delivery for specific needs (frequently related with health). Inside this ecology of services is where our service has to survive and be useful, ethical and provide a service to individuals and community. On the one hand, users at the end of the day will choose to hire the services of a person or have a support system installed, with all possible hybrid solutions. On the other, synergies help to provide a better global service, together with market optimization, or success. A later section gives some examples of such synergies.

A critical issue found is social relation of the direct users. We have found directors of shelter homes that didn't want any technology in their rooms because of the threat of decreasing the little social relation their clients have, often related with their dependence and cares. We were also told by social workers that some elders receive fewer visits as soon as a tele-alarm is installed. On the other hand, our very first project, Ashored, gave us an example of transforming relations from dependency to peer to peer, when grandchildren came to play with the new computer of grandparents and as a consequence of feeling alive again we could hardly find them at home after the project to perform some follow up because they started to travel. In a nice the way home technology boosted them out of home into better peer-to-peer relations.

Thus we have come together to consider the social dimensions that should be taken care of not as an absolute burden, but to carry out some preventive and promotion actions to always leave the person in a better situation as it was before services came to his/her home.

Another last point refers to the framework seen from this dimension. It is translated into new specifications, as limitations of sustainable cost or use of infrastructures that institutions maintain. In line with the former issue of interaction among services, we received a firm limitation of having just one set-top box per home, to take care of leisure, health and AAL considerations, otherwise our local government could not support project actions.

## 4. Results and Discussion

We have structured the service description tool in four categories that generate the information that we found relevant in service information sharing: user-oriented, technology-oriented, market-oriented and social and institutional context. As information items are already covered, we give here an example of service description, as in our experience it is an optimum way to make previous arguments more understandable. As stated, our teams have been traditionally focused on technology developments, user requirements and evaluation, so the two information sets generated by the users-team and technology team are the ones that we detail most.

The others have come from the need to attend to company-market or free-market sustainability (in open-ware style, as worked with special education schools and special occupational labor centers for people with disabilities) and from numerous meetings with responsible local government politicians and technical staff in which their goals, requirements and limitations, parallel application fields of interest, decisions about using public structures, combination of two or more departments in the promotion of a set of services, *etc.*, were brought up, sometimes too late in the design process.

### Description of an AAL Service: Night Falls and Wandering Prevention

The service is focused on the elderly that get up during the night to go to the bathroom with poor lighting. It is oriented to a home scenario and consists of detection of getting up and itinerary and timing to check the person comes back to bed without difficulty. Dimmed lighting is controlled and if a potential risk situation is detected, a relative or professional staff is notified. This service has been implemented in two homes of elderly people and in some rooms of a shelter home and it has also been worked out in several creative sessions in master studies in AAL.

### 4.1. User-Oriented Features

The first section of ZAAS-DT is user-requirements. In our development we chose a classic method, user-fit, which we have used in simplified versions in the different development projects mentioned.

Through the experience of using this tool with several collectives in the frame of three different master studies in technical aids for gerontology, we have found a repeated lack of comprehension of some differences among items of this tool, so we have modified it to make it simpler to use, and hopefully clearer. Several iterations in the simplification have been offered to different generations of students (mostly social workers, nurses, medical doctors working directly with the elderly in one or another fashion), gradually incorporating their feedback in successive revisions.

#### 4.1.1. Identification of User Profiles and Needs Regarding their Special Capacities and Main Goals

Primary users:
  BENEFICIARIES: Elders that may suffer confusion or falls when getting up in the middle of the night.
  CARERS: Relatives.
Secondary users:
  Service Center
  Installation and maintenance
Quaternary users: local governments

Some examples of needs related to each user category are shown in Table 5.

#### 4.1.2. Identification of Scenarios and Associated Requisites

Scenario is home-like. Table 6 shows examples of analysis of requirements coming from the use in different rooms.

As the scenario is indoors, at home, no special ambient requirements are needed. If it were to include a garden, for instance, devices would have different requirements regarding humidity protection and operating temperature range.

#### 4.1.3. Identification of Activities and Associated Requisites

In this case activity is walk and going to the toilet and the system only will consider the time employed in them. The user can start other activities at night: such situations will be treated as time-out and warn the user directly to check if going back to bed is the option, with the freedom of the user of inactivate the supervision. Table 7 shows examples of this other source of specifications.

#### 4.1.4. Functional Specification from User Context

Table 8 compiles functional requirements of the service coming from the previous points, getting user requirements grouped.

Here we can detect possible conflicts and state priorities, in the way user-fit methodology indicates.

Specifications in this table are mainly oriented to drive the design and later evaluation of the system. The team will enter in an iterative process with technology in which functional specifications translation into technological specifications will reveal alternatives, difficulties and new opportunities. For this process, it is important to take care of team good relations so that others perspective is understood as a source of enrichment and not a competence. Also in this phase we have found burdens with vocabulary and with differences in priorities. Information in this table will be later completed by market and institutional considerations and goals.

#### 4.1.5. Human Machine Interface (Requisites Coming From User Context)

Specifications for Human Machine Interfaces (HMIs) will have at least two entries, one coming from the user's capacity and the other in combination with technological possibilities and design ideas. What follows is an example of Human Machine identified interfaces and examples of user's requirements related to them:

‐ HMI_user:
  Local. May be needed in several home rooms and activate the closest one to the user.
  Perceptual features: low vision; acoustics at night and potential low hearing.
  Cognitive features: Not many items, very simple navigation if needed.
  Interaction features: elderly regular capacities.
  Information and interaction: will warn an anomalous situation is detected and check if user is confused giving him/her the opportunity to stop warning (configurable to adapt to confusion level of users).
‐ HMI_couple or relative living with the main user:
  Local. Can be the same physical device as for the HMI_user. May be needed in several home rooms and activate the closest one to the user.
  Perceptual features: low vision, acoustics at night and potential low hearing.
  Cognitive features: not many items, very simple navigation if needed.
  Interaction features: elderly regular capacities.
  Information and interaction: system will advise care-giver an anomalous situation is detected giving basic information: place and time passed for user detection.
‐ HMI_reference relative living in another house (relative of reference):
  Remote on the mobile for relative.
  Perceptual features: regular vision; regular hearing; acoustics at night.
  Cognitive features: regular.
  Interaction features: regular.
  Information and interaction: system will advise relative an anomalous situation is detected giving basic information: place and time passed for user detection. It may give the possibility to open an infrared camera and check if user is ok if permissions and ethics are positive.
‐ HMI_care center: Similar to relative HMI, notifications will go to the care-center instead of relative's mobile.

#### 4.1.6. Innovation and Acceptance Expectations (User Context)

We did several interviews and questionnaires to elderly living in couples and alone, caregivers in shelter home and relatives of elders. The result is that they show interest in a system like the one described.

### 4.2. Technology-Oriented Features: System Description and Modules

#### 4.2.1. Graphical and Modular System Description

**Figure 6.**
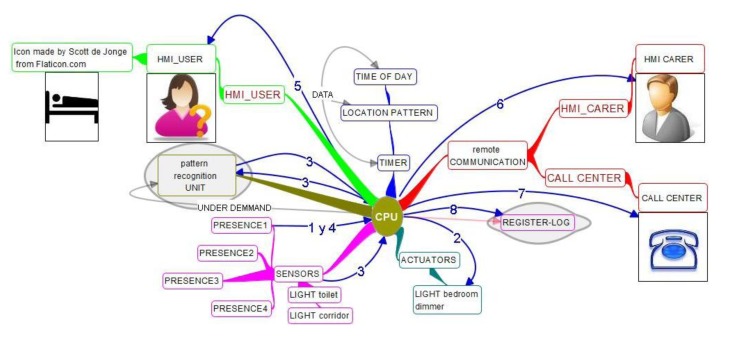
Modular system description for night falls and wandering prevention.

The modular composition of the system is described in Figure 6. It has a Central Processing Unit (CPU) with some interoperable middleware (*i.e.*, OSGi—Open Service Gateway initiative). It has movement sensors for detection of getting up from bed, corridor walking, toilet entering or staying, and presence in other home rooms. It has some pattern recognition calculation unit to calculate where the person is and learn about habits, paths and timings.

It actuates with the light of the bedroom to have it on to prevent falls. It gets data such as time of day, location pattern, and timer of staying in each stance. It may also need calendar or other additional data. Specific additional modules not shown could be light actuators on the corridor to show the path; camera in specific rooms.

#### 4.2.2. Information Flows

Information flows are important so everyone understands which type of information is flowing where and at what time. It also shows the sequence of possible actuations, mapped onto technological modules, so it is a previous and more understandable step than algorithm for non-technological people to understand the service functioning. It is shown in Figure 6:

1. System detects getting up from bed and looks into information of presence sensors and light switches sensors.
2. System turns on dimmed light on bedroom.
3. System provides information to pattern recognition unit and starts timing the presence in each room.
4. If user returns to bed without any time out, service goes back to check getting up sensor.
5. If a time out is found, the display closest to user will try and get a response reminding it is time to sleep and offering to stop notification to care-giver. User may stop the notification and this will reset all timers. User may stop notifications and also may not stop them.
6. If user doesn't stop notifications care-giver HMI is used to notify situation.
7. Notification action may be sequential to different recipients until one is answering and takes action on the situation.
8. All actions that are considered significant are registered to offer adjustment parameters to singular environment and use and also statistics of false negatives and positives for further improvement and for political strategic decision taking.
9. Optionally, a remote care-giver may ask for further information to the system (even open a camera), as the time when the user got up.

#### 4.2.3. Modules and Functions Dependency Tables

Some of this information is presented in previous graphics. Table 9 includes software modules and organizes information so as to facilitate identification about which technological modules are supporting each function.

In case of redefinition of project goals, this table has proven useful to check the priorities of development of moduli to keep important functions and identify which other could be dropped with not too large functional implication.

#### 4.2.4. Human Machine Interface

Table 10 shows functions to be covered by interfaces with the moduli in which they are to be implemented and technical functions planned. Users may check easily how displays and interfaces are being designed and keep an eye in specifications being fulfilled as they understand (detect conflicts in understanding) and also keep an eye in new possibilities that selected devices may give at almost no additional cost.

#### 4.2.5. Maturity and Cost of Each Module

From common sense and as also proven in project experiences, when real demonstrators are to be deployed technology modules need to be in good and reliable shape. Table 11 shows how our consortium described the status of the different moduli development and estimated cost for budget adjustment prevision. In cases when the status is commercial, another simple table would keep track of suppliers and procurement logistics.

#### 4.2.6. Functional Description: Algorithm

**Figure 7.**
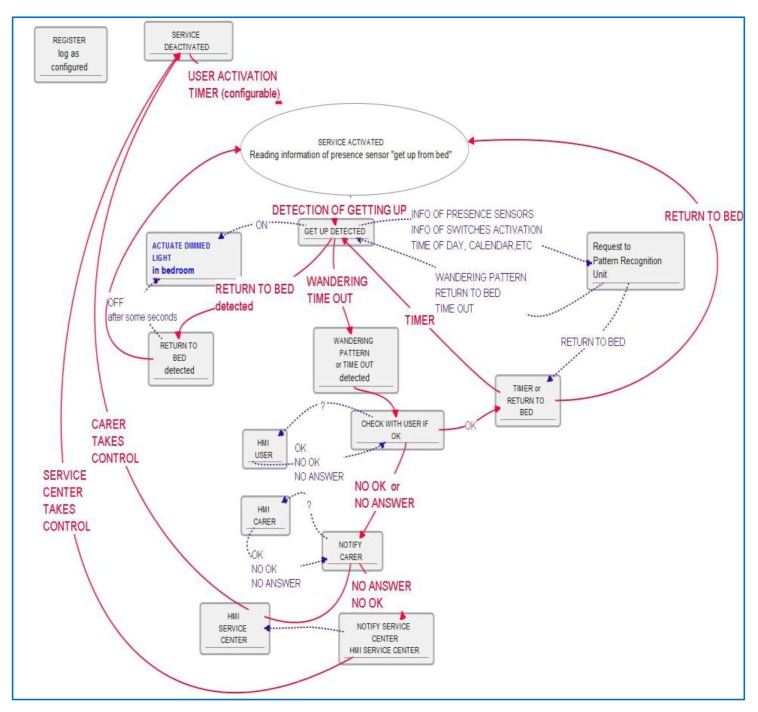
Simplified algorithm for night falls and wandering prevention; actions flow is in red lines; information exchanging is in blue-dotted lines.

Once sequential functions and moduli are clear for non-technological professionals, we come closer to technological language description with the algorithm description, as shown in Figure 7.

Little additional explanation about delays, states and transitions are needed to complete understanding and then complete functionality planned is accessible to users, again being able to check functionalities and promote new ideas or configuration parameters may be they didn't think of at the first stage. This “understanding-along” process is found very rich to get new functions or personalize service parameters.

### 4.3. Market-Oriented Features

#### 4.3.1. Added Value and Market Innovation

Market research prior to any investment is crucial, as companies and institutions have shown firmly in our experience in past projects and in the internal guidelines they have provided.

A service should add enough innovation features to make it attractive by market. Our service has some functionalities and features that will be detailed regarding this market expected attraction. Then a comparison with other systems on the market is to be performed and the resulting advantages or our service highlighted. If result is that such service exists, or it does in too similar way, considerations of adopting such other system are to be made, and otherwise consortium needs to have very clear the advantages of this particular action prior to investments. Innovative market features can be crucial for service success, so they have to be incorporated in design with corresponding priority level. Table 12 gives a prior idea of this simplified study.

#### 4.3.2. Sustainability and Costs

Taking the information of production costs, market adds distribution, installation, maintenance, *etc.*, provides the users and institutions figures about sustainability, linked with information about options to modify financial schemes. This market side is done by corresponding departments in companies or by market experts. When coming to institutions, this in linked with economic impact.

#### 4.3.3. Value Chain

**Figure 8.**
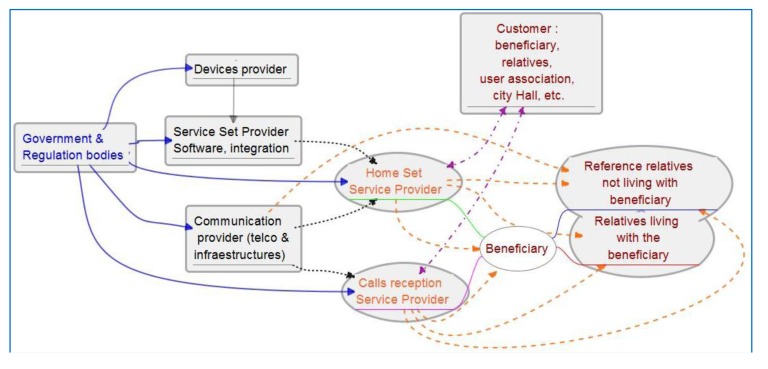
Service provision chain of services that include calls to service centers: In blue font (outgoing full blue lines) quaternary stakeholders; black (dotted) tertiary, orange (dashed) secondary and red font primary. Violet lines (dash-dot) show contractual and financial relation.

Figure 8 shows a scheme of the service provision chain for our service. Colors distinguish primary, secondary, tertiary and quaternary users, and arrows of the corresponding colors show the service provision chain. In the case of quaternaries it is regulation. There is a potential difference between customer and beneficiary, although they can be the same person. Customer is understood here as the one signing the service contract and paying for it (violet lines). It can be a relative of the elders, user organization or even the city hall. There may be other contractual relations between the city hall and the final beneficiary. Table 13 shows the study of other existing services or structures which can interact or support the new service. Interaction may occur because of coincidences in service provision chain, using common structures, similar financing concepts, adding new functionality to existing services, *etc*.

#### 4.3.4. Financial Paths

**Figure 9.**
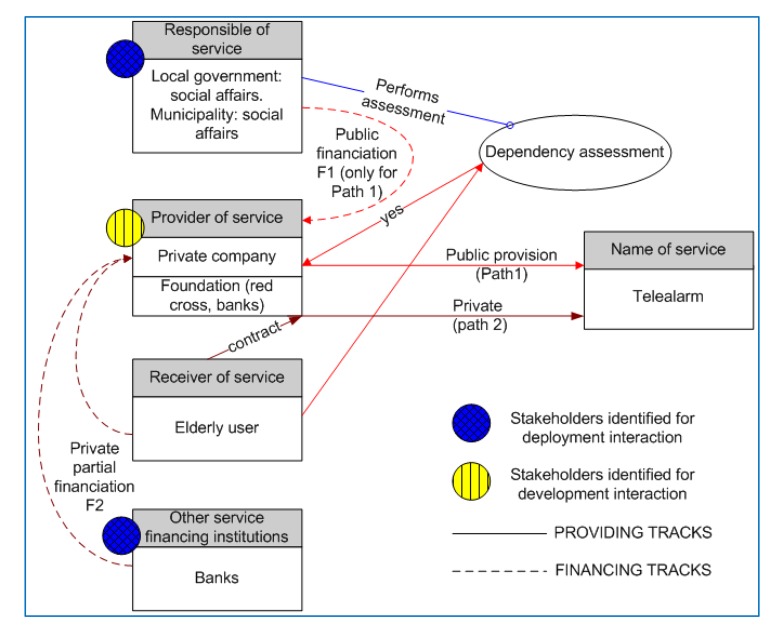
Financial and provision paths for service using service center.

In Figure 9 financial paths are shown, together with service provision paths. It shows the ways in which a user can enter both service provision paths and financial paths (granted some financial help or classified as eligible for standard financial support). Ways of entrance are showing how users enter via public financing or privately, and normally are linked in this AAL field to diagnosis by medical doctors, detection in any other service or user's or relative's demand. In other projects with children, for instance, entrance path can be through the observation of a primary teacher who detects abnormal capacities in a child and asks for evaluation.

#### 4.3.5. Public Economy Impact

Impact on the public economy is not a straightforward study, yet it is essential in many AAL services efficiency and sustainability studies. Such services often imply quality of life of economically non-profitable sectors of population. When there is profit, private companies will probably do it.

Public institutions need to see which ways and alternatives there are to use money efficiently. Fall prevention may have a large impact on public health costs, from the extra domestic costs of having an elderly person with reduced mobility and implications for the social security costs of their carers to medical attention, surgery, prosthesis, *etc*.

Early fall attention will also have an impact on such costs. A studied balance of such costs may show that small investments will save large amounts of money, further improving life quality of population sectors.

#### 4.3.6. Domestic Economy Impact

AAL services are based on technology. Nevertheless when looking into solutions for a home there are many ways and even combinations, as proven by the different programs set up by Non-governmental Organizations (NGOs) and administrations: neighbors, trans-generational exchange programs, hiring the services of a person.

Different alternatives need to be quantified and offered to the final user so the decision about which alternative suits better each situation is finally taken by the end user.

### 4.4. Social and Institutional Context-Oriented Features

#### 4.4.1. Institutional Additional Goals

Institutions need specific information or features of the system to decide whether or not finance a service in the ecosystem of alternatives and better serve the citizen. In this service it is needed to know the success ratio of detection and user acceptance, so a new feature of the system is the register of incidences that will allow a simple study of efficiency and use of the service.

#### 4.4.2. Security, Legal and Ethical Issues

Specifically any information that may come out from the system has to be protected, as well as any other that may remain in the system as register data. Data protection laws throughout Europe are becoming stricter to better protect citizens. Other issues considered are insurances and product regulations to be on the market.

#### 4.4.3. Existing Services with which it Interacts and Can Combine

As a complement to Table 13 made from a market perspective, conclusions from institutions may be to merge services that use public financed structures to improve efficiency. In our experience this is to be cared for carefully, especially if they may imply more than one public administration department or any additional role for an actor. Sustainability depends sometimes on this specific matter. For this service, tele-alarms are to be considered for merging in the same pack, together with other similar services.

#### 4.4.4. Support Structures with which it Combines

This service uses Service Centers as specific service provision structures. It also uses internet access or telephony access, which is taken care of by technological information bits.

#### 4.4.5. Potential Social Impact and Sociogram

This feature is shared by user-professionals. This service may be adapted to people living alone by recruiting neighbor volunteers. As being normally a time when users sleep, not many new relations or affection of existing ones is expected. Otherwise the expected or resulting sociogram [33] and the one prior to intervention give clues regarding which social relations could be promoted and/or transformed.

#### 4.4.6. Institutional Goals Functional Specification

As a summary, we can summarize institutional dimension features in some functional specifications. The two following ones are examples of specifications we have received:

System must have a log which allows study of efficiency of the system and is compliant with data protection regulations.

System is to be combined to tele-alarm service centers. If proven successful, it has to be prepared to be merged with tele-alarms and also exist as an independent service.

### 4.5. Illustration Diagrams for Supervision Services

In order to give a vision of how ZAAS-DT is used for different services, functional diagrams are shown for a service of supervision of activity and home status aimed for AAL support for the elderly with need of light supervision. Part diagrams and tables are:

#### 4.5.1. Graphical and Modular System Description

**Figure 10.**
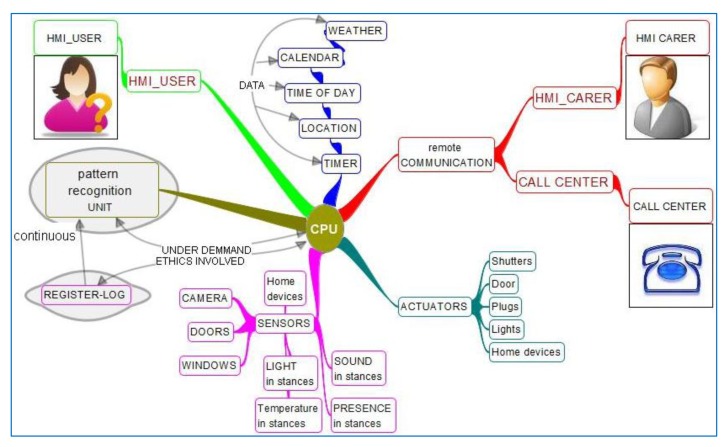
Modular system description for activity and home status supervision service.

Modular composition of the system is described in Figure 10.

The main structure is similar to former services, where basically there is a Central Processing Unit (CPU) with some interoperable middleware that allows data processed by any application to be available to any other, together with the possibility to connect sensors, actuators and information processing units from any vendor or provider that gives connectivity information. This service has more complete information in the way of available data for applications, for pattern recognition of home status and activity may need to be referenced very many items, from calendar or time of day to time of food intake and user mood. The sensors also provide much more information. In order to allow for automatic response, or remote support provision such as a relative helping with shutters or any other automation, the diagram shows different common actuators available on the market. Communication is again facilitated with a relative or caregiver as well as with a call center. Also, a log and post-processing of information about use and contextual information is provided, both to adjust configuration, update and analyze patterns dynamically and obtain use and efficiency statistics to provide grounds for investment and political decisions to relevant stakeholders.

Information flow in this case is to be set in accordance to pattern recognition, ethics and capacity and preferences of the user. Typically sensor information will be directed continuously to a pattern recognition unit which in turn will start events, incidences or alarms as programmed. Security alarms will go directly to the outside, as potential intrusion detection; activity deviations from patterns will typically go through user supervision before going out, with a time out in case user is confused or unable to manage, giving the user the possibility to change behavior privately, depending on his/her capacities.

This modular diagram will be completed or simplified to adapt to real situations of dwellings. Significance in supervision can be obtained with only light sensors, or only presence sensors, temperature sensors or any others.

#### 4.5.2. Functional Description: Algorithm

Functional definition of this service can be really elaborated, for there are very many parameters that can be taken into account in contextual information, and deviations from patterns vary significantly in their importance (from critical to recommendations to check) depending on user capacities and preferences.

**Figure 11.**
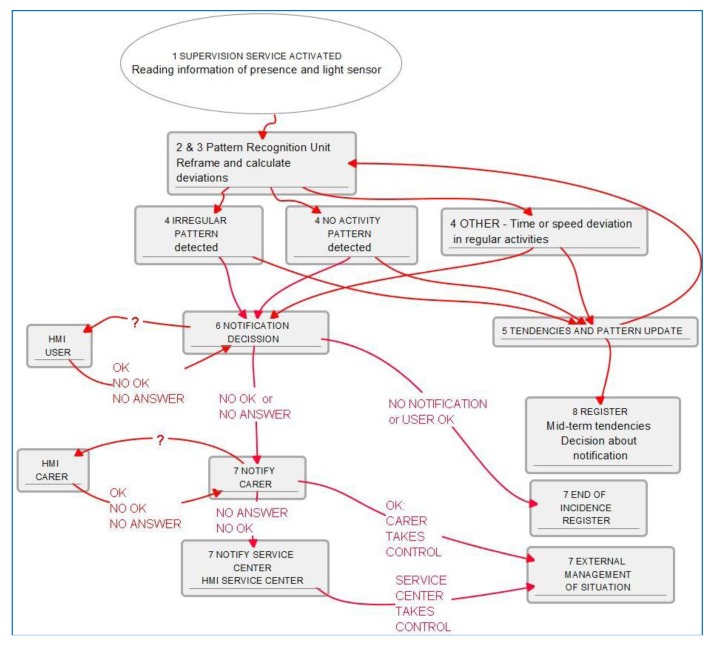
Supervision service simplified algorithm.

In order to provide further illustration we describe a simplified algorithm for presence and movement detection patterns and light patterns in rooms, illustrated in Figure 11, which can easily show regular activity in a daily timetable, modulated by calendar and user mood and planned activity. This paper's aim is not to choose an algorithm description, so we will show here a textual functional description, also used in our projects when functions are simple enough and to resume blocks of functions:

1. Information reading from presence and light sensors.
2. Framing information to calendar and internally to reference day activities (references considered to be included for common understanding of stakeholders).
3. Calculating light patterns and deviations with the re-framed timetable. Calculating movement and presence patterns and deviations.
4. Decision taking about the significance of deviations, classification of potential results: potential critical issues such as no movement in the house and no light in the living room once the day is advanced; incidences such as delayed food intake or slower movements through the house.
5. Input of results in deviations in patterns tendencies, which will allow for pattern updating and tendencies finding which may mean progressing into life-styles changes.
6. Decisions about notifications: to the user to raise awareness of tendencies to help regulate them into healthier life-styles, to the user and later to care-giver if deviations are deemed critical or tendencies need to be looked after.
7. Management of detected incidences.
8. Register of incidences, result and tendencies. Check-up of maintained tendencies and detection of potential medium-term deviations. Decisions regarding notification.

#### 4.5.3. Service Provision Chain Diagram

**Figure 12.**
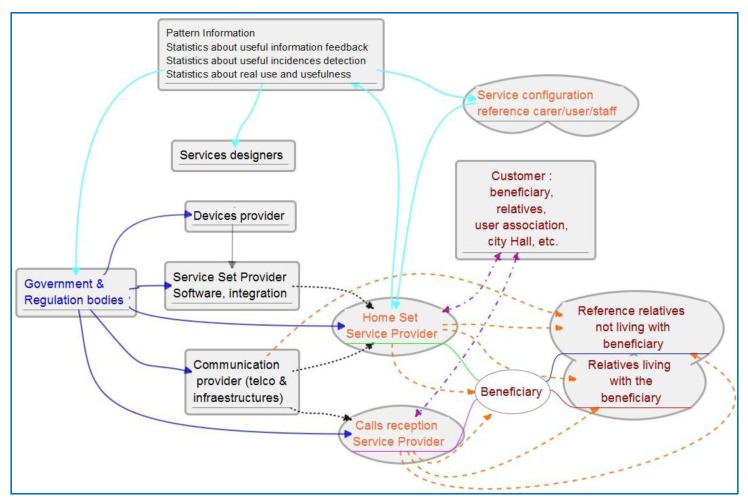
Service provision chain and pattern use for reconfiguration, redesign and regulation bodies information.

Figure 12 shows the service provision chain for the supervision service. It shows additionally how supervision information is also information about the usefulness of the system for re-design and regulatory agents and also for re-configuration or adaptation of functionality. In such collaborative ecosystems every agent accesses information to better play the role it has in the community to keep improving AAL support to the focus population.

## 5. Conclusions/Outlook

A service description methodology and set of diagrams and tables for structured information sharing called ZAAS-DT has been proposed to assist in the collaborative design of AAL environments and services. This tool was born from the need to promote understanding of global service scenario to all collaborating sectors in its definition, development and deployment.

One service example is shown together with diagrams of a second one for better understanding information structure and sharing capacity. Both are classical examples of services in AAL for the elderly. It provides for an inclusive perspective for different types of actors and stakeholders that the authors haven't found in literature, being this its basis for innovation. It tackles the difficulty of sharing understanding of crucial issues related to services design and implementation from different professional perspectives. Each professional type generates specific information that is shared aiming at a common understanding inside a consortium, with the goal to achieve a more complete, efficient and sustainable service for the entire community. Underneath it the shared priority of a global benefit including actors found relevant in service design and sustainability *versus* defending partial views and opinions is understood. This methodology intends to make operative the will to give value and understand others' views and collaborate promoting globally optimized solutions.

It has defined four dimensions for the AAL field. Users and technology are more developed than the market and social or institutional dimensions. Currently our team is designing works to include the market dimension with more implication.

In this paper, evaluation design hasn't been described, although it can also be considered a part of ZAAS-DT for it adds and uses information bit sharing. Anyhow the summary goals together with requirements will give the information that later will be checked by evaluation.

The description tool has proved efficient for collaborative purposes in several European and regional projects showing its positive benefits: it serves as a communication repository, so different professional groups can access information generated by the others. It also reminds consortia of the need of global benefit prioritization aiming for a global scope *versus* partial views, helping the steering of the project.

Based on difficulties to solve in developing services in collaborative way, this tool addresses:

*Vocabulary*: It helps in vocabulary conflicts, for the explanation, graphics and tables give enough detail to check whether the understanding has been common or not.
*Stating service requirements*: Requirements are added up in a structured and shared way by all four stakeholders, including other dimensions considerations that could escape to a singular group of professionals.
*Keeping user requirements in the design loops and cases of use*: Enhancing understanding of components of a service and their implications, *i.e.*, for taking steering decisions: it also helps in such common understanding to have access and understanding to important information needed to take collaborative decisions that take into account a complete perspective from different partners. In this way, it helps having arguments behind decisions clear and explicitly stated. This covers maturity and capacity of reaction to unexpected results: implications of moduli delay or drop-off, information flows and system sequential functioning, data protection and quality of service in each information flow, relevance of each module in the overall performance of a service.
*Service contextual information as critical for sustainability*: Especially it is found useful when tackling service sustainability beyond the project or specific financial support and identifying and taking advantages of synergies with other existing services in the community and technological scenarios.

Moreover, it promotes understanding and boosts collaboration by providing a common ground in which conflicts and further opportunities are identified, as new functionalities at little extra cost or further configuration parameters of the service to adapt it to different user's situations. This way, a consortium can take advantage of both conflict detection and opportunity identification.

## Figures and Tables

**Table 1.** Description of importance of information generated by user-oriented professionals as received by other professionals.

**Info-Bits By Users**	**Other Professionals**	**Description**
User profiles needs and goals	Technology Market Institution	Main goal of R&D in technological assistive services is to improve assistance to users by solving their needs. Profiles and user needs are leading the technological proposal, market sustainability and social function of institutions.

User scenarios, activities and requirements	Technology	Design of technology has to follow studied needs. Technology people not only need this info available, they usually need to contrast understanding, and scenarios and activities give them very good keys to understand the casuistic and promote good solutions.

	Market	Market orientation will definitively depend on user profiles, scenarios and activities in scope.

Functional specification	Technology	Understanding functional specifications is a must for successful design of technological services. Normally an interaction should occur among users and technicians so functional specifications can be met and many times improve by the suggestions of available technical solutions.

Innovation and expected acceptance	Market	Commercial feasibility of a product or service will largely depend on the acceptance of potential clients. Felt innovation is a good clue to orient market strategy. Other parameters as will of buying the service are also essential.

**Table 2.** Description of importance of information generated by technology-oriented professionals as received by other professionals.

**Info-Bits by Technology**	**Other Professionals**	**Comment**
Technological specifications	Users	Specifications feedback is crucial so users can understand that their functionalities are covered and technicians can make sure the way they have understood it. By iteration, technicians may propose different solutions to check which cover user needs and improve reliability, cost or any other feature.
Graphical modular system description	Users	Participation of users is crucial throughout the process, so specifications are kept or modified according to needs. These images help understanding how the system works without much depth or complexity, and we have found this understanding is enough to take project global decisions. Users also may identify expensive or reliable uncertain moduli and assess relevance of keeping them as it will impact the overall cost of the system.
Information flow	Users	Information flows for each service helps the understanding of its designed dynamics. It also helps check out critical information treatment, as privacy issues and quality of service in each information path.
Cost and maturity of each moduli	Market	Market can make estimations of cost and preview stages in which different functional subsets will be reliable following the project calendar. It also will make suggestions of which moduli should be prioritized to have those subsets ready.
	Institution	Institutions will look into which investment is to be made, and adapt it to the priorities and specific needs. It also will be used to compare with alternative solutions.
HMI description	Users	Users here will check that needed features of HMI are understood and followed, and sometimes it is found they were not well-defined, so the project may be in time to re-design such HMIs before too much effort is invested into them (we use the quality criteria of ISO/IEC 9126: Software Engineering Product Quality Model [14]).
Technological support structures	Institution	We have found that regular communications technology is needed in the service ecology and often institutions are responsible for them. Many times it only takes a political decision in time to facilitate this issue, so institutions should be made aware as early as possible of such needs, because in our experience it may be the slowest agent collaborating in a project.

**Table 3.** Description of importance of information generated by market-oriented professionals as received by other professionals.

**Info-Bits by Market**	**Other Professionals**	**Description**
Innovation and added value	Technology	Innovation and added value are important arguments for market introduction and sustainability. In such perspective, design receives this information as additional specifications, completing or prioritizing the existing ones.
Costs chain and sustainability	Technology	Cost horizon is crucial for sustainability, so again this becomes an important specification for it imposes restrictions on the technological solutions that can be used.
	Institution	To obtain a cost-map that gives good sustainability prevision to the services, initial and maintenance costs of a service have to be taken into account by institutions to calculate the investment and guarantee it will be sustainable in time.
Other competing Services	Institution	On one side selection among feasible alternatives is needed. On other side, interaction with existing services may give collaboration opportunities that give better or cheaper overall service to the citizen or may result in cost reduction.
Potential financial chain	Institution	Often financial chains are affected by public institutions, which may change the balance between cost and interest by grants or subsidies. Having a clear map will show if financial paths or the modifications that can be made on their structures will give sustainable results.	
Impact on public economy	Institution	Political responsibility makes institutions and society very much aware of the overall cost of elder and disabled people well-being. Impact may show savings where in principle only initial costs are visible.
Impact on home-economy	Users	The same as before, applied to individual or family-group decisions.

**Table 4.** Description of importance of information generated by institutions as received by other professionals.

**Info-Bits by Institutions**	**Other Professionals**	**Description**
Security, ethical and legal issues	Users	Guarantee of personal data protection, security and respect for individual rights of users of the system are a must and increase the confidence in the services. Moreover, it is a compulsory requisite and as such is to be checked by users and included in specifications.
	Technology	This guarantee is included in specifications that will define the constraints and options over the system so the complete ecology of actors may decide on the final result regarding these issues.

Interacting existing services	Market	Some services depend on different administration departments and are built on top some other public or private services. Such dependencies bring the need of permissions or modifications of public services requirements, and may be crucial for market survival and sustainability of services. Political decisions over such conflicting or interacting services open or close market opportunities, so the market needs to be well aware of such perspectives to make realistic market strategies.

Sociogram and potential impact	Users	In our experience any support service that has been introduced in a living environment has some impact on the social relations of the users, from some elder whose grandchildren started visiting much often thanks to the games in the computer when there were not so common as today, to other situations in which visits are diminished because of the tranquility of knowing the person's security is supervised. Other examples are people who took care of their personal hygiene and image for they had a daily interview to check how things were; also people who started to have closer relation with relatives thanks to Skype-like communication in their shelter home. Compensatory actions can be taken when social deterioration is expected.

Functional specification	Technology	Institutions sometimes include some specifications on the services that don't exactly correspond to user needs, in terms of cost, difficulty of installation or maintenance, staff who can be taking new roles to name but a few. These specifications are to be included in design ones.

**Table 5.** Analysis of user needs and system requirements regarding user capacities.

**User profile (Elder)**	**User singularities that may affect system use**	**System requirements**
Elderly living alone or with couple	Potential unstable walking Potential confusion episodes Potential low vision at night	Detect situations that could evidence falls or confusion. Turn on a dimmed light on the itinerary to help orientation and decrease falls risk

Care-Giver couple	Potential low vision	Acoustic interface and/or large display

Care-Giver relative	Conventional, may live far	Remote notifications needed

Service Center staff	Regular service center for tele-alarm	Telephone-wired communication

Local government	Interest in impact of such system	Register incidences to check usefulness of the system

Installation and maintenance	Installation has to be done in a particular home.	Optimum if service can be installed as a kit by user's relatives

**Table 6.** Analysis of user needs and system requirements regarding scenarios.

**Scenarios for Beneficiary (Home)**	**Scenario Singularities That May Affect System Use**	**System Requirements**
Bed-room at night	If living in couples, dimmed light shouldn't bother the couple.	Luminosity of dimmed light adjustable. Position of dimmed light adjustable
	Amount of light needed may vary	
	Sensors of getting up shouldn't detect couple instead	Position of movement sensors needed adjustable, so area of sensitivity has to be adjustable.
	When getting up in the middle of the night it is difficult the person remember to wear anything on	System should work with the user wearing no special device

Corridor at night	Would improve some dimmed light.	Distribution of movement sensors and dimmed lights in the corridor, only if severe confusion.
	In cases of disorientation, optimal spaced lights would help keep path	

Bathroom at night	Risk of falls is higher in the bathroom	Useful to detect when light is being turned on, so system can time and check when user goes out or raise an event of too long stance.

Any other stance of the house at night	User has the privacy and right to wander around his home. Confusion may make him/her wander and forget it is time to sleep	System should detect excessive periods out of bed and warn. Warning will address the user directly or the caregiver if confusion level is very high.

**Table 7.** Analysis of user needs and system requirements regarding activities.

**Activities for Beneficiary (Elder)**	**Activities Singularities that may Affect System Use**	**System Requirements**
Walk or go to the bathroom	Privacy required.	System will time activities and warn the user if too much time is gone or he/she stays in other rooms longer than established.
	Checked by time out.	
	User could decide to start an activity, even being at night	
		System can be deactivated by the user or couple/relative if user is very confused.

**Table 8.** Service functional requirements.

**Identification of the Service**			
FALL/CONFUSION PREVENTION/DETECTION AT NIGHT			
List of Actors	List of scenarios	List of activities	
1. Elderly; 2. Care-Giver couple; 3. Care-Giver reference relative; 4. Service Center staff; 5. Local government	Bedroom, corridor, bathroom Any other home stance	Walk Go to toilet	
Service needed features	Rational	Priority	Conflicts/ needed actions
Detect situations that could evidence falls or confusion	Potential unstable walking and potential confusion episodes of primary user	Very high	–
Turn on a dimmed light on the itinerary to help orientation and decrease falls risk	Potential low vision at night of primary user	high	–
Acoustic interface and/or large display for HMI	Potential low vision of primary user and couple	high	–
Remote notifications needed	Conventional, may live far	high	–
Telephone-wired communication	Regular service center for tele-alarm	high	–
Register incidences to check usefulness of the system	Interest in impact of such system by local governments	medium	–
Luminosity of dimmed light adjustable	Not to bother couple	Medium	–
Position of dimmed light adjustable	–	–	–
Position of movement sensors needed adjustable, so area of sensitivity has to be adjustable.	Not to cause false positives by the movement or getting up of couple	Medium	–
List of Actors	List of Actors	List of Actors	
System should work with the user wearing no special device	User capacities may make it difficult to wear on something when getting up at night	high	–
Distribution of movement sensors and dimmed lights in the corridor, only if severe confusion	For users that are very confused	low	–
Useful to detect when light is being turned on, so system can time and check when user goes out or raise an event of too long stance	To time activities and know when the user is active in toilet	Very low	–
System should detect too long stances out of bed and warn. Warning will address the user directly or the caregiver if confusion level is very high	Risk of fall active or confusion situations	high	–
System will time activities and warn the user if too much time is gone or he/she stays in other rooms longer than established	User could decide to start an activity, even being at night. First check with user, then if no answer given, caregiver will be informed	High	–
System can be deactivated by the user or couple/relative if user is very confused	For special days, system can be de-activated at user's own risk	High	–

**Table 9.** Modules supporting each function.

**Items/Functions**	**Software**	**Devices: S for Sensor, A for Actuator**
Detect situations that could evidence falls or confusion	Timer of presence in a stance	S_presence
	Pattern recognition of normal/abnormal situation	S_Switches

Turn on a dimmed light on the itinerary to help orientation and decrease falls risk	Decide when light is to be turned on/off	A_light_dimmer

Register incidences to check usefulness of the system	Log	–

Luminosity of dimmed light adjustable.	Adjust light intensity to ambient light measurement	S_light_level
Position of dimmed light adjustable		A_light_dimmer

Position of movement sensors needs to be adjustable, so the area of sensitivity has to be adjustable	–	S_presence (adjustable in sensitive area)

System should work with the user wearing no special device	–	–

Distribution of movement sensors and dimmed lights in the corridor, only if severe confusion	Inference of position with presence sensors	S_presence
	Decision which light to turn on/off by checking inferred position with presence sensors	A_light

Useful to detect when light is being turned on, so the system can time and check when the user goes out or raise an alarm about an event of excessive duration.	–	S_switches

**Table 10.** Analysis of requirements for Human Machine Interfaces (HMI).

**Function**	**Device**	**Technical Functions Needed**
Acoustic interface and/or large display for HMI	User_HMI: Small tablet fixed in different places at home	Acoustic / visual information. Tactile interaction
Remote notifications needed	Carer_HMI: Remote communications module	Sms and internet messaging
Telephone-wired communication	Carer_HMI: Wired telephone communication module	Wired telephone modem

**Table 11.** Analysis of maturity and cost of each module.

**Module**	**Maturity**	**Estimated Cost**
S_presence	Commercial	80 euros
A_light_dimmer	Commercial	80 euros
S_Switches	Prototype. Ready for production	40
S_light_level	Commercial	40
A_light	Commercial	60
S_presence mechanical visor	Prototype. Ready for production	10

**Table 12.** Added value and innovation.

**Features**	**Our Service**	**Other Service**	**Relevance or Market Opportunity**
Detects getting up from bed	√	–	How this is done can be crucial
No need to wear or carry any device by user	√	–	Very important for usability and acceptance
Detects wandering patterns	√	–	Only in cases confusion is a threat
Detects time outs: provides assistance if falls	√	–	Check upon wellbeing of client
Lights dimmed light to prevent falls and disorientation	√	–	Very important prevention action
sequential recipient notification	√	–	It can save service center fee
Option to user to stop notifications	√	–	It can avoid false positive errors and protect privacy of user

**Table 13.** Analysis of interaction with existing services.

**Services**	**Potential Beneficiaries. Service Locus**	**Responsible Institution Funding**	**How to Access to Public Funding**	**Service Provider**	**Additional Benefit of Service**
Night wandering and falls prevention/supervision	Elderly people in independent living scenarios at home	Local Government—Social Affairs—Dependency Program Local municipal Social Services Financing available from both	Private	Proposal:(specific company or NGO: Non-governmental Organization)	Innovative alert situations, direct call to relatives. Provide for passive alarms, integration in a middleware platform will provide benefits of integration with other services in later phases Innovative monitoring functions; integration with most frequent local support systems. Can take profit of existing structure for tele-alarm and service centers
Municipality Tele-Assistance	Elderly people with acute and/or chronic disabilities. People with cognitive disabilities At home	Municipal Social Services Financing available Financing also by banks	People need to be assessed as having a need by local authority for public financing; otherwise can be obtained privately	Private company by public contract (names of companies providing tele-alarms)	
